# Systems-level analysis of the plasticity of the maize metabolic network reveals novel hypotheses in the nitrogen-use efficiency of maize roots

**DOI:** 10.1093/jxb/erab522

**Published:** 2022-01-05

**Authors:** Samuel M D Seaver

**Affiliations:** Argonne National Laboratory, Data Science and Learning Division, Argonne, IL, USA

**Keywords:** Genome-scale metabolic modeling, nitrogen-deficient stress, maize root, metabolic shift, development, metabolomics

## Abstract

This article comments on:

**Chowdhury NB, Schroeder WL, Sarkar D, Amiour N, Quilleré I, Hirel B, Maranas CD, Saha R.** 2022. Dissecting the metabolic reprogramming of maize root under nitrogen-deficient stress conditions. Journal of Experimental Botany **73**, 275–291.

**Alleviating the overuse of nitrogen fertilizer may compromise crop yields, and understanding the efficiency with which plants utilize nitrogen will help us engineer crops to maintain yields while using less fertilizer. As the assimilated nitrogen is necessary for the biosynthesis of many components of plant biomass, there is interest in studying the nitrogen-use efficiency (NUE) of crops in a systemic manner. In their article in this issue of *JXB*, Chowdhury *et al.* (2022) analyze the behavior of the metabolic network in the maize root as it responds to growth in nitrogen-poor conditions. The authors reconstructed the metabolic network of the maize roots *in silico*, enabling them to integrate both transcriptomics and metabolomics data sampled from roots of maize seedlings grown under either nitrogen-rich or nitrogen-deficient conditions. Crucially, they run simulations on the metabolic network using the integrated data to constrain the outcomes, and examine how reaction fluxes and metabolite pools vary between the two conditions.**

## Nitrogen deficiency in maize

The demand for a chemical source of nitrogen as a fertilizer for all the world’s crops is unsustainable, partly due to the environmental impact of using fossil fuels to drive the synthesis of fertilizer and partly due to the negative effect of dumping large quantities of fertilizer on native ecosystems (Udvardi *et al.* 2015; Wang *et al.*, 2021). Reducing the amount of fertilizer while maintaining the yield that would feed the world’s population has been an important goal and there is burgeoning research into the efficiency with which crops utilize nitrogen (Li *et al.*, 2017; Kant *et al.*, 2011). It was estimated that a 20% increase in NUE would reduce the nitrogen requirements by 1.4 Mt year^–1^ (Langholtz *et al.*, 2021). In order to examine the relationship between the use of fertilizer and the yield of the crops, researchers are growing crops in nitrogen-poor conditions and examining the mechanisms by which nitrogen is assimilated (Amiour *et al.*, 2012). The responses are not only observed in the transporters and subsystems that assimilate N directly; they ripple across the primary metabolic processes that produce biomass as part of the plant’s development and growth, impacting the eventual yield, and highlighting the need for a system-level phenotype to characterize them (Kant *et al.*, 2011).

## Genome-scale reconstruction of maize tissue

The reconstruction and simulation of the entire metabolic network encoded in the genome of any species has been an important tool in understanding the systemic manner in which an individual may respond to perturbations in its environment. The reconstructions are the result of the extensive collating of enzymes and the biochemical reactions they catalyze from the available data in databases and the literature. Genome-scale metabolic reconstructions for plants have been built and rebuilt since the first published models of the core central carbon networks for *Arabidopsis thaliana* (Poolman *et al.*, 2009) and *Chlamydomonas reinhardtii* (Boyle and Morgan, 2009) in 2009. A recent review highlights the number of species for which these reconstructions have been built (Tong *et al.*, 2021). The reconstructions are dubbed ‘genome-scale’ because authors strive to include all the enzymes encoded within the sequenced genome of the species in question. However, the enzymes are all differentially expressed in different tissues and under different conditions, and several approaches have been made either to reduce the size of the networks so that they are specific for the tissue in question (Seaver *et al.*, 2015) or to alter their simulated behavior (Machado and Herrgård, 2014). In every approach, this requires the integration of omics data, and this is the case of the work described here.

## Metabolic reprogramming is a systemic response

The reconstruction of the genome-scale metabolic network of the maize root *in silico* allowed the authors to examine the systemic impact of the nitrogen-limiting growth regime on the root tissue. Some of the same authors previously published a similar study on the NUE in the maize leaf, with the data being sampled from several different developmental stages during the plant’s growth (Simons *et al.*, 2014), and the authors are now focused on performing some of the same analyses in the maize root. Much of this work is focused on the manner in which the network is built, is simulated using the integrated data, and how the approach is validated by comparing the experimental pooling of metabolites with their theoretical results. One novel approach that the authors take here is to extend the flux-sum analysis approach (Chung and Lee, 2009; Lakshmanan *et al.*, 2015) to simulate a proxy for the range of concentrations at which a metabolite may pool under the environmental conditions, which they call flux-sum variability. By using this approach, they highlight eight lipids, and two precursors (methionine and citicoline), whose metabolic pool sizes were not directly coupled to the biosynthesis of biomass components, and hypothesize that they play a role in the increased growth of roots that occurs under nitrogen-poor conditions.

## Systems-level responses extend beyond tissues

The metabolic network that was reconstructed for any tissue can be combined with those reconstructed for other tissues and organs in the same species. As the authors have already generated the metabolic network for the maize leaf, a natural step forward in this work would be to connect the networks representing the root and leaf tissues, via the means of a metabolic network representing stem tissue, so as to combine the sources of assimilated nitrogen and carbon, respectively. Whole-scale plant tissue modeling has already been implemented and advocated by several groups (Grafahrend-Belau *et al.*, 2013; Shaw and Cheung, 2018; Gomes de Oliveira Dal’Molin *et al.*, 2015). For the individual tissues, these sources are modeled as fixed inputs from the ‘environment’, either sucrose for the root or amino acids for the leaf, but by combining the two metabolic networks for each tissue and allowing the abundance of these metabolites to be dynamically constrained by the integration of omics data, researchers may reach additional conclusions on how the mechanisms of nitrogen assimilation and carbon assimilation may interact.

## Are there key metabolic shifts during development?

The conclusions in this work were reached using data that represent a single snapshot during the development of maize seedlings. For the purpose of the study, half of the maize seedlings were grown in nitrogen-poor conditions until sampled at the 6- to 7-leaf stage and, as such, the reprogramming in question had occurred over the course of two and a half weeks. While the catalysis of the enzymes in the network occurs within very short time scales, the spatial and temporal pooling of key metabolites can occur over longer time scales. The pooling of these metabolites may in turn impact the regulation of the metabolic network via several means, but could also do so unevenly, where different metabolites pool at various rates, affecting metabolic subsystems at different stages of the plant’s development. Large-scale sampling of these metabolites at these developmental stages may reveal the timing of these metabolic shifts, but the generation of such datasets is expensive and time-consuming, particularly as the data are noisy, and the researchers may not know in advance when to sample the tissues. The work performed here could be done in combination with other approaches such as dynamic flux balance analysis (Mahadevan *et al.*, 2002; Flassig *et al.*, 2016; Shaw and Cheung, 2018; Schroeder and Saha, 2020) in order to guide this approach, to discover when these metabolic shifts occur, and improve the temporal targeting of the engineering for NUE.
